# Supplementary material for: Huntingtin CAG-expansion mutation results in a dominant negative effect
Source: Front Cell Dev Biol. 2023 Sep 1;11:1252521. doi: 10.3389/fcell.2023.1252521 (PMC10505792; doi:10.3389/fcell.2023.1252521)
Supplement: Supplementary file 1 [file DataSheet1.pdf]

SUPPLEMENTARY FILES

Huntingtin CAG-expansion mutation results in a dominant negative effect

Tiago L. Laundos[1,2,3,4], Shu Li[1], Eric Cheang[1], Riccardo De Santis[1], Francesco M. Piccolo[1,†,*] and Ali H. Brivanlou[1,†,*]

[1]Laboratory of Synthetic Embryology, The Rockefeller University, New York City, New York, United States

[2]ICBAS - Instituto de Ciências Biomédicas Abel Salazar, Universidade do Porto, Portugal

[3]i3S - Instituto de Investigação e Inovação em Saúde, Universidade do Porto, Portugal

[4]INEB - Instituto de Engenharia Biomédica, Universidade do Porto, Portugal

†Last authorship: These authors share last authorship

*** Correspondence:**

Francesco M. Piccolo
fpiccolo@rockefeller.edu

Ali H Brivanlou
brvnlou@rockefeller.edu

Keywords: Huntington's disease (HD), Huntingtin (HTT), human embryonic stem cell (hESC), Dominant negative, Organoids.

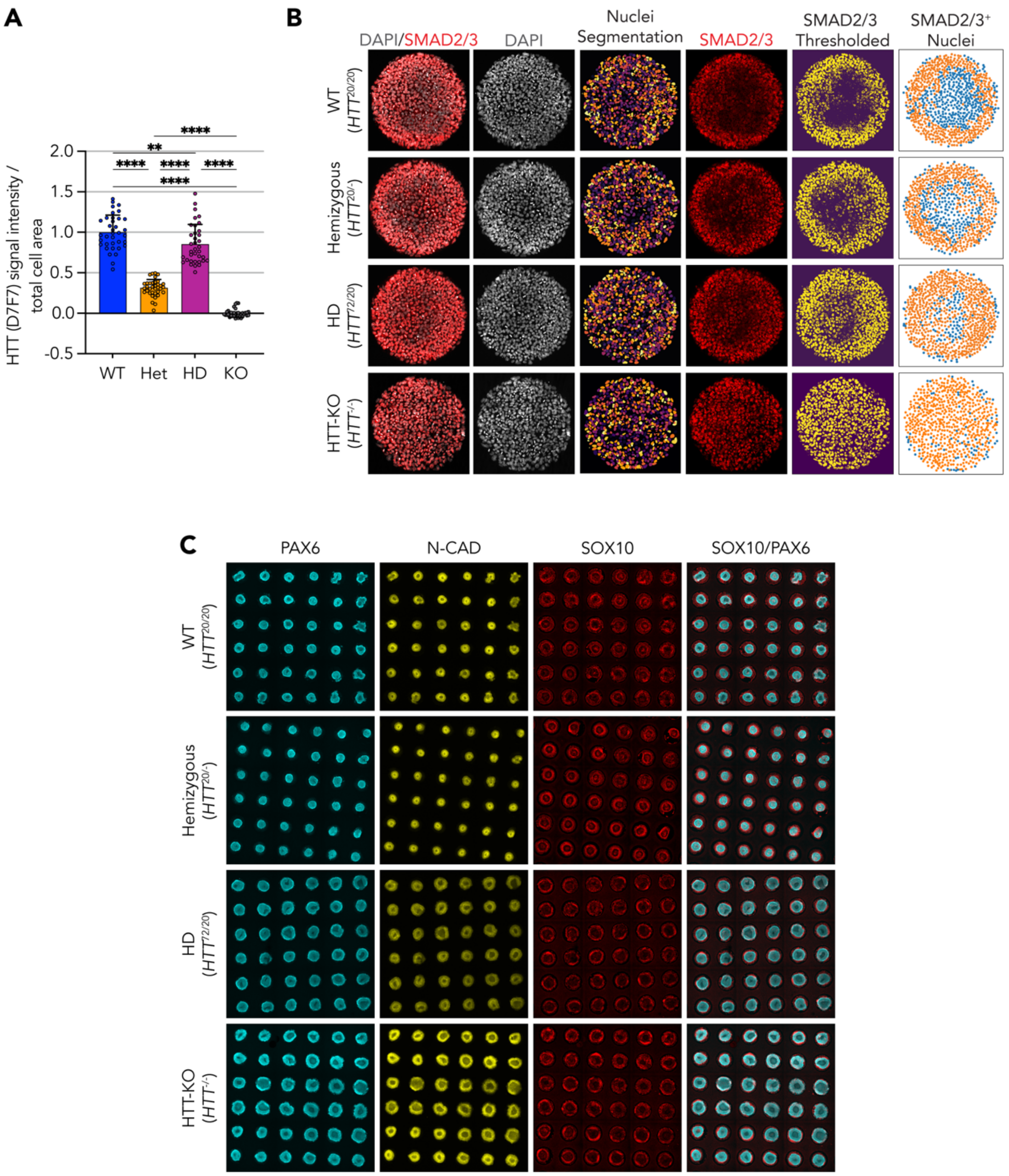

Figure S1 A Quantification of immunofluorescent signal of HTT (D7F7 antibody) reveals halving of HTT amount in the Heterozygous cell line, compared to WT and HD cell Line; signal density was normalized to area occupied by ESC colonies (n=36 fields). **B** Pipeline for quantification of Fraction of SMAD2/3$^+$ nuclei; Segmented individual nuclei were matched with their integrated SMAD2/3 density and classified as positive (orange) and negative (blue) after manual thresholding. **C** Overview of the micropatterned cultures used in the neuruloid assay show the range of variability between individual colonies observed in this assay.

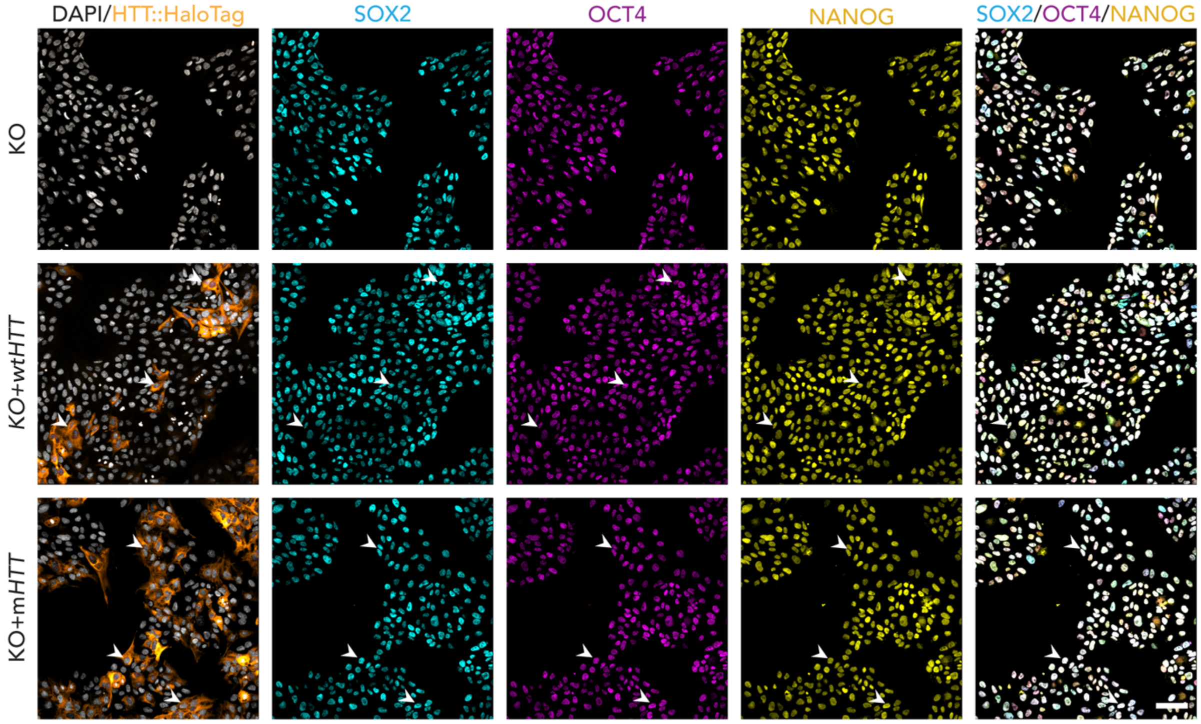

Figure S2 - HTT-KO cell line carrying wt or m*HTT-HaloTag* transgene retain co-expression of pluripotency markers SOX2, OCT4 and NANOG. Scale bar: 100μm.

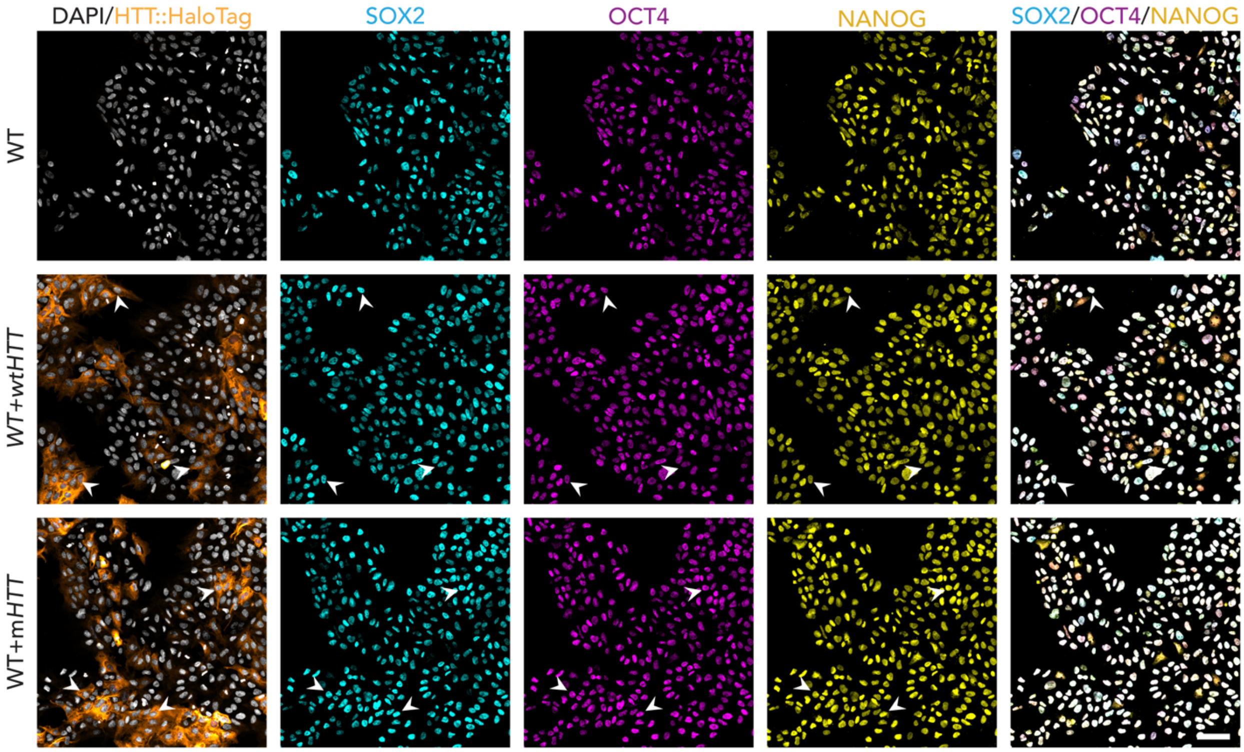

Figure S3 - WT cell lines carrying wt or m*HTT-HaloTag* transgene complementing endogenous *HTT* levels retain co-expression of pluripotency markers SOX2, OCT4 and NANOG. Scale bar: 100μm.

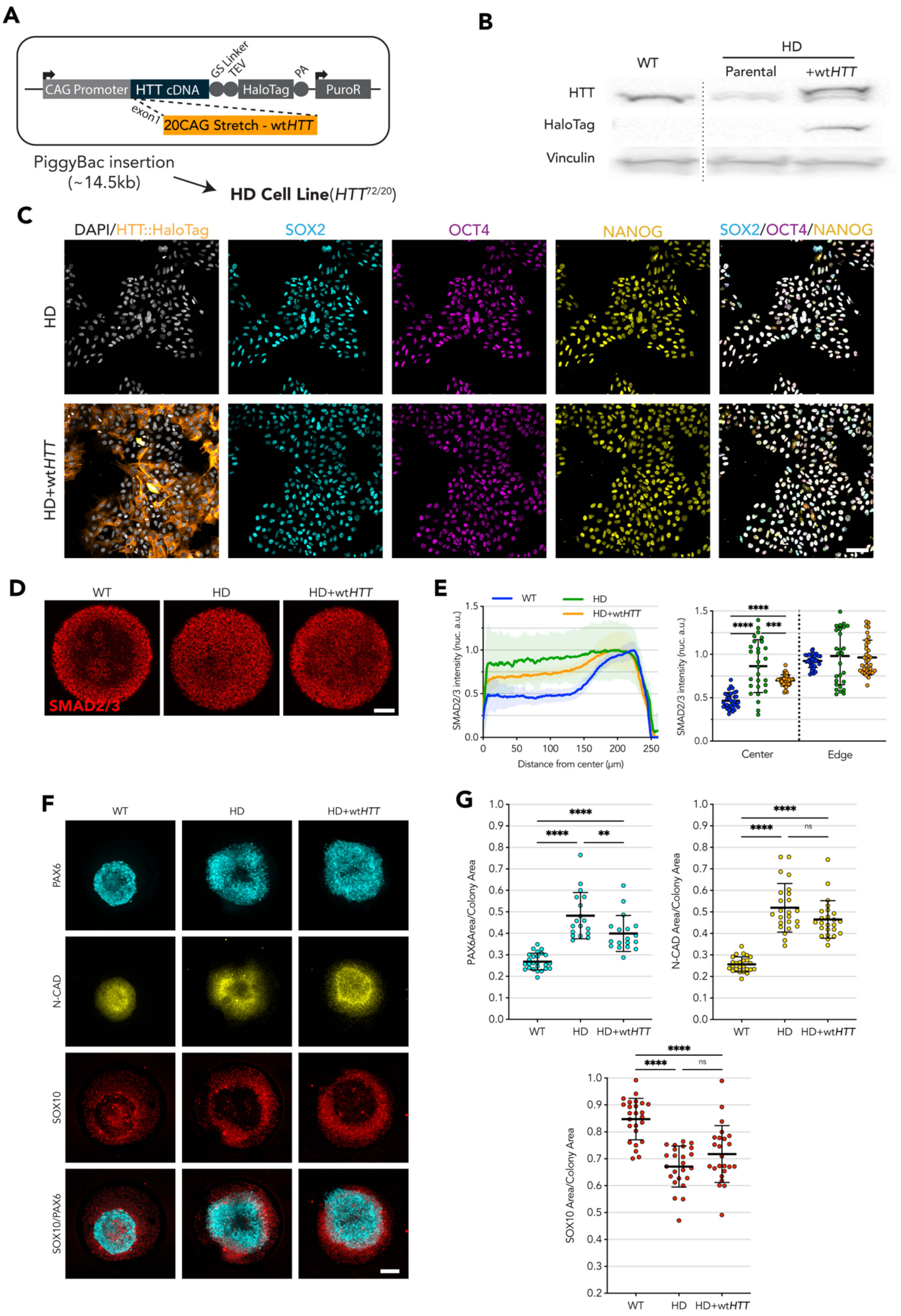
A
CAG Promoter
HTT cDNA
GS Linker
TEV
HaloTag
PA
PuroR
exon1
20CAG Stretch - wtHTT
PiggyBac insertion
(~14.5kb)
HD Cell Line(HTT72/20)
B
WT
HD
Parental
+wtHTT
HTT
HaloTag
Vinculin
C
DAPI/HTT::HaloTag
SOX2
OCT4
NANOG
SOX2/OCT4/NANOG
HD
HD+wtHTT
D
WT
HD
HD+wtHTT
SMAD2/3
E
WT
HD
HD+wtHTT
SMAD2/3 intensity (nuc. a.u.)
Distance from center (μm)
Center
Edge
F
WT
HD
HD+wtHTT
PAX6
N-CAD
SOX10
SOX10/PAX6
G
PAX6Area/Colony Area
N-CAD Area/Colony Area
SOX10 Area/Colony Area
WT
HD
HD+wtHTT
ns

Figure S4 – wt*HTT* transgene expression partially rescue the phenotype in HD cells A Puromycin selectable ePiggyBac transposon carrying full-length wt*HTT* was inserted into a HD cell line (bi-allelic 20CAG/72CAG background), complementing endogenous expression of wt and m*HTT*. **B** Immunoblot against HTT and HaloTag confirms the stably integrated wt*HTT-HaloTag* construct in the Parental HD line. **C** HD (*HTT*$^{20/72}$) cell lines presenting wtHTT-HaloTag recombinant protein expression complementing endogenous *HTT* levels retain co-expression of pluripotency markers SOX2, OCT4 and NANOG. **D** Immunofluorescence imaging shows SMAD2/3 nuclear translocation upon Activin A stimulation, limited to the edges on WT and Het colonies with wider to full induction on the colony center HD and to a lesser extent in HD+wt*HTT* colonies. **E** Mean radial intensity profile of nuclear SMAD2/3 shows insensitivity to Activin A at the colony center in HD+wt*HTT* is rescued, albeit partially (scatter plot displays the mean SMAD2/3 nuclear intensity for each colony at center:25-10μm and edge:175μm-225μm; WT n=34, HD n=28, HD+wt*HTT* n=36). **F** Representative immunofluorescence images of Neuruloid induction assay performed on 500μm circular micropatterns. **G** Complementation of HD hESCs with wtHTT transgene resulted in a reduction of PAX6 area, showing partial rescue of the phenotype (WT n=25, HD n=24, HD+wt*HTT* n=24). Groups were compared using one-way ANOVA followed by Dunnett's *post-hoc* test for correction of multiple comparisons (* $p<0.05$, ** $p<0.01$, *** $p<0.001$, ****$p<0.0001$, ns $p>0.05$). All values are presented as mean ± SD. a.u.: arbitrary units. Scale bar: 100μm.

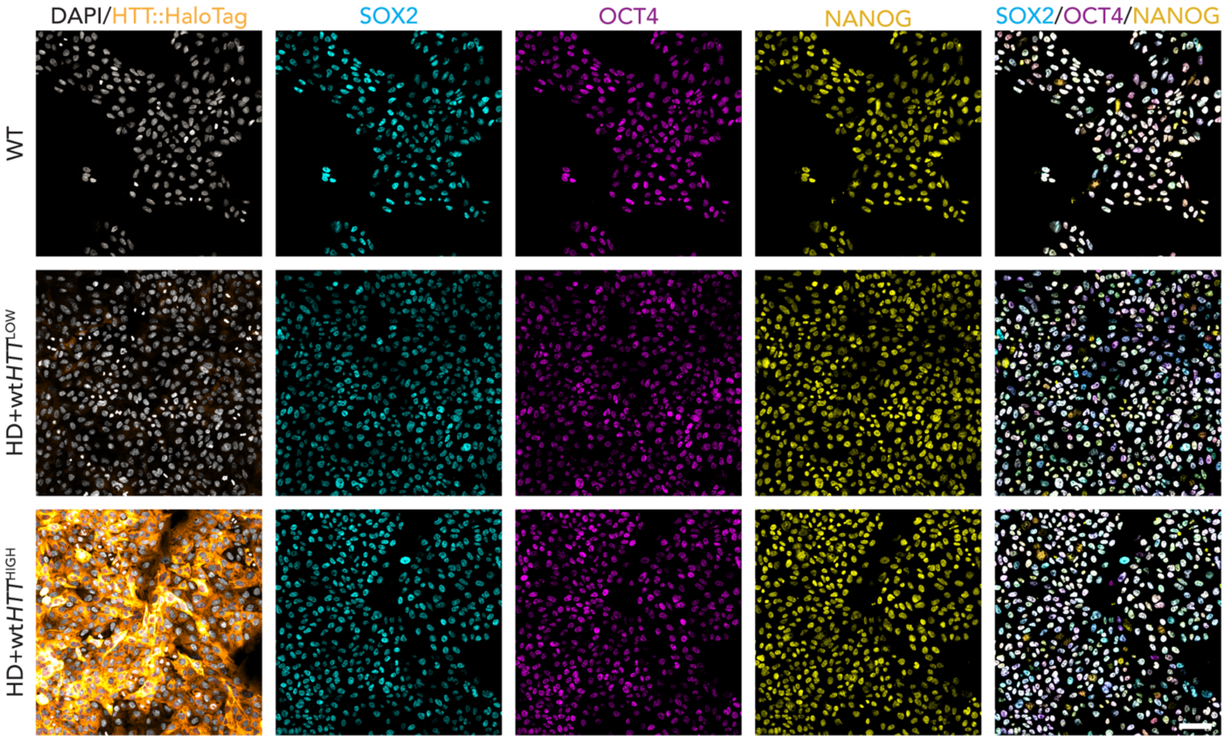

Figure S5 – HD (*HTT*$^{20/72}$) cell lines presenting wtHTT-HaloTag recombinant protein at Low and High levels after FACS, retain co-expression of pluripotency markers SOX2, OCT4 and NANOG. Scale bar: 100μm.

Antigen	Antibody	Application	Dilution
**HTT**	Cell Signaling Clone D7F7 Rabbit mAb Cat no. 5656	Immunofluorescence	1:100
**HTT**	Millipore Clone 1HU-4C8 Mouse mAb Cat no. MAB2166	Immunoblot	1:1000
**Vinculin**	Millipore Clone V284 Mouse mAb Cat no. 05-386	Immunoblot	1:2000
**HaloTag**	Promega Rabbit pAb Cat no. G9281	Immunoblot	1:1000
**PolyQ-HTT**	DSHB Clone MW1 Mouse mAb Cat no. MW1	Immunoblot	1:1000
**SMAD2/3**	BD Biosciences mouse mAb Cat no. 610842	Immunofluorescence	1:100
**PAX6**	BD Biosciences Clone O18-1330 Mouse mAB Cat no. 561462	Immunofluorescence	1:300
**NCAD**	BD Biosciences CD325-PE mouse mAB 561554	Immunofluorescence	1:200
**SOX10**	R&D Systems Goat pAb Cat no. AF2864	Immunofluorescence	1:50
**SOX2**	R&D Systems Goat pAb Cat no. AF2018	Immunofluorescence	1:200
**OCT3/4**	BD Biosciences Clone 40/Oct-3 Mouse mAB Cat no. 611202	Immunofluorescence	1:200
**NANOG**	Cell Signaling Clone D73G4 Rabbit mAb Cat no. 4903S	Immunofluorescence	1:200

Table S1 – Antibodies used in this study.
